# Alteration of Th17 and Treg cells in patients with unexplained recurrent spontaneous abortion before and after lymphocyte immunization therapy

**DOI:** 10.1186/1477-7827-12-74

**Published:** 2014-08-03

**Authors:** Li Wu, Li-Hua Luo, Ying-Xin Zhang, Qing Li, Bo Xu, Gui-Xiang Zhou, Hong-Bing Luan, Yu-Sheng Liu

**Affiliations:** 1 Center for Reproductive Medicine, Department of Obstetrics and Gynecology, Anhui Provincial Hospital Affiliated to Anhui Medical University, Hefei, Anhui, China

**Keywords:** Th17 cells, Treg cells, Unexplained recurrent spontaneous abortion, Lymphocyte immunization therapy

## Abstract

**Background:**

Several types of T cells have been associated with the pathogenesis of unexplained recurrent spontaneous abortion (URSA), including Th1/Th2/Th17/Tregs cell. It has been appreciated that immunotherapy with paternal or third party lymphocytes is an effective method of treatment for URSA patients. The balance of Th1/Th2 cells could be maintained and an increase of Treg cells would be beneficial after immunotherapy; however, the mechanism by which the Th17/Treg balance affects URSA has not yet been fully elucidated.

**Methods:**

Here, we used flow cytometry, liquid chip technology and quantitative real-time PCR (qPCR) methods to characterize Th17/Treg cell populations after immunotherapy. We found that after immunotherapy in URSA patients, the percentage of Th17 cells decreased and the percentage of Treg cells in peripheral blood mononuclear cells (PBMC) increased, as detected by flow cytometry.

**Results:**

Immunotherapy may induce a decrease in the Th17/Treg ratio and the Treg bias, which may be beneficial for the maintenance of pregnancy. The expression level of ROR gamma t, a transcription factor found in Th17 cells, decreased and the expression of the Treg-specific transcription factor Foxp3 increased in peripheral blood as detected by qPCR. Immunotherapy may induce a decrease in the ratio of ROR gamma t to Foxp3 and a Treg cell bias, which would be beneficial for pregnancy maintenance. The secretion of the Treg-associated cytokine TGF-beta, as well as Th2 cytokines, was increased in serum, while the secretion of Th17-associated cytokine IL-17A and Th1 cytokine production was decreased. The Th1/Th2 cytokine ratio significantly decreased. Similarly, the Th17/Treg ratio significantly decreased in the total patient after immunotherapy.

**Conclusions:**

These results indicate that in patients with URSA, immunotherapy with mononuclear cells derived from the baby’s father could affect both Th1/Th2 and Th17/Treg balance, and we found that the Th2 and Treg bias would be beneficial for pregnancy, which may lead to a balancing of the Th17/Treg ratio in URSA patients after immunotherapy.

## Background

An estimated 1–3% of women have experienced three or more consecutive miscarriages prior to 20 weeks gestation, and this is defined as recurrent spontaneous abortion (RSA) [1]. Unexplained recurrent spontaneous abortion (URSA) affects approximately 50% of RSA patients, and it is largely associated with the failure of feto-maternal immunologic tolerance. The embryo expresses paternal antigens that are foreign to the mother and therefore may be viewed as an allograft; in normal pregnancy, the embryo is not rejected by the mother’s immune system [2]. Literature indicates that altered immunity in URSA is dominated by what is known as the Th1/Th2 hypothesis [3]. The emerging concept of the Th17⁄Treg balance has challenged the conventional paradigm of Th1⁄Th2 hypothesis [4,5]. Th17 and Treg T cells have been described as two subsets that are distinct from Th1 and Th2 cells with opposite effects on autoimmunity and transplantation tolerance [6,7]. Lee SK et al. [8] reported that the imbalance between Th17 and Treg cells was correlated with the pathogenesis of URSA. Several reports have suggested that the normal human pregnancy is associated with an elevation of the immunosuppressive Treg subset, and Tregs were recognized to play a crucial role in the maintenance of normal immune tolerance [9-11]. Wang et al. [12] and our previous findings [13] demonstrated that the percentage of Th17 cells were higher in the peripheral blood of patients with URSA, suggesting a potentially negative role of Th17 cells in the maintenance of pregnancy.

Since the 1980s, immunotherapy with paternal or third-party lymphocytes has been an effective treatment for URSA, and it effectively allows for the suppression of the maternal immune responses to the allogeneic fetus in vivo during pregnancy [14-18]. The mechanisms by which immunotherapy works have not yet been fully elucidated, but it may skew the balance of Th1/Th2 cells in patients with URSA who undergo immunotherapy [19-21]. Several studies have indicated that lymphocyte immunotherapy significantly increased the proportion of CD4+/CD25+ Treg cells in peripheral blood mononuclear cells (PBMCs) in women with URSA, and it ultimately improved pregnancy outcome [22]. Several reports found that Th17 cells may play a vital role in rejecting conceptus antigens and therefore may be detrimental for the maintenance of pregnancy. An abnormal balance of Th17/Treg cells may exist in URSA [8,12,13]. However, the association between immunotherapy and Th17 cells and the Th17/Treg balance has not yet been fully elucidated.

In this study, we found a decrease in the proportion of Th17 cells in PBMCs and decreases in the expression of Th17-related cytokine IL-17A and Th17-related transcription factor RORγt mRNA [23] in peripheral blood after immunotherapy with paternal or third party lymphocytes. Furthermore, an increased proportion of Tregs in PBMCs as well as elevated expression of Treg-related cytokine TGF-β and Treg-associated transcription factor Foxp3 mRNA [24] were also detected in the peripheral blood. We also found that the Th17/Treg cell ratio was decreased and that the Treg bias improves pregnancy outcome following immunotherapy. After analyzing successful pregnancies that had undergone immunotherapy, we found that the Treg cell bias contributed to the improvement of pregnancy outcome. Taken together, these results help clarify the etiology of immunotherapy with paternal or third party lymphocytes and provide a novel target for the prevention or intervention of spontaneous abortion via regulation of the Th17/Treg balance.

## Methods

### Patients

The study was conducted in compliance with the Declaration of Helsinki and Ethics Committees on Human Research of Anhui Provincial Hospital, an affiliation of the Anhui Medical University. This study had the approval of the Ethics Committee of Anhui Province Hospital; informed consent was obtained before samples were collected (2010 Ethics 5th). All the subjects were patients of the outpatient department of gynecology at Anhui Province Hospital (Hefei, China) between May 2010 and May 2011. Through communication with all couples, they agreed to participate in the study and signed an informed consent form prior to enrollment. This is a prospective study. Twenty patients who had experienced at least three confirmed successive spontaneous early abortions (7–12 weeks of gestation) of unexplained etiology with one partner were recruited to participate in the study. The mean ± SE deviation age was 28.6 ± 0.15 (ranging from 25–37) years, and the median number of miscarriages was 4 (range, 3–7, and all the women had natural conception). None of the participants had a genetic impairment, Mullerian anomaly, hormonal deficiency, metabolic disorder, infectious disease, or autoimmune abnormalities, such as positive antiphospholipid antibodies or lupus anticoagulant, in our systemic workup. In addition, tests for thrombophilic status, such as protein C activity, protein S activity, and thrombin anti-thrombin III complex were routinely performed for all patients (Details were showed in Additional file 1: Table S1).

All patients were healthy except for their history of recurrent abortions and were negative for blocking antibodies, which were identified by a one-way mixed lymphocyte culture reaction (responder: patient, stimulator: husband), according to previously reports. Berifly, Lymphocytes were collected from heparinized blood via Ficoll-Hypaque gradient centrifugation. The mitomycin C-treated stimulator cells of the husband and responder cells of the patient were mixed and co-cultured in RPMI 1640 containing either pooled human AB serum or tested serum for 6 days. After a pulse time of 18 hours cultured with 3H-thymidine, the cultured cells were harvested and the DNA synthesis was evaluated by liquid scintillation counting. The blocking effect (BE) was calculated as following BE = (1-meancpm of culture in tested serum/mean cpm of culture in AB serum)*100 (%).

### Paternal or third-party lymphocytes immunization

Donor (husband or third party) lymphocytes were prepared by Ficoll-Paque centrifugation; the cells were washed three times with sterile saline and resuspended in 1 ml at a concentration of 2–3 × 107 cells/ml. The cells were administered three times intradermally at 3-week intervals. We collected the blood after fourth immunization (about 12 weeks after first immunization), and all samples were collected at the same time point. In this study, the lymphocyte immunization therapies were performed every 3 weeks for four times, and then maintaint the lymphocyte immunization therapy every 6 weeks.

### Cell preparation

For Th17 analysis, human PBMCs were suspended at a density of 2 × 106 cells/mL in complete culture medium (RPMI 1640 supplemented with 100 μ/ml streptomycin, 100 u/ml penicillin, 2 mm glutamine and 10% heat-inactivated fetal calf serum (Gibco, Invitrogen, CA, USA)). The cell suspension was transferred to 24-well plates. Then, cultures were stimulated with phorbol myristate acetate (PMA, 50 ng⁄mL) plus ionomycin (1 μg/ml) for 5–6 hr in the presence of monensin (500 ng⁄mL; all from Alexis Biochemicals, San Diego, CA, USA). Cells were grown in a 37°C incubator at 5% CO2. After 5–6 hr of culture, the contents of the wells were transferred to 5-mL sterile tubes. The cells were then centrifuged at 350 g for 5 min. For Treg analysis, PBMCs (100 μL) were aliquoted into tubes for further staining.

### Surface and intracellular staining

Cells were aliquoted into tubes and washed once in phosphate buffered saline (PBS). For Th17 analysis, the cells were incubated with fluorescein isothiocyanate (FITC) anti-human CD4 (eBioscience, San Diego, CA, USA) at 4°C for 20 min. For Treg analysis, the cells were incubated with PerCP anti-human CD4 (eBioscience, USA) and FITC anti-human CD25 (eBioscience, San Diego, CA, USA) at 4°C for 20 min. After the surface staining, the cells were then fixed and permeabilized with Perm/Fix solution (Beckman Coulter) and were stained with PE anti-human IL-17A (eBioscience) for Th17 detection or PE anti-human Foxp3 (eBioscience, CA, USA) for Treg detection. All staining was performed according to manufacturer’s protocols. Isotype controls were used to enable correct compensation and confirm antibody specificity. Samples were analyzed using a FACS Calibur flow cytometer and Cell Quest Pro software (Beckman Coulter).

### Quantitative real-time PCR (qRT-PCR)

The tissues were collected in RNase-free tubes and total RNA was extracted using Trizol reagent (Invitrogen). RNA samples were quantified with a NanoDrop™ 1000 Spectrophotometer (Thermo scientific) and electrophoresis. Briefly, the peripheral blood cells were homogenized in Trizol reagent, and then chloroform was added to the 1/5 volume of Trizol and mixed thoroughly. The mixture was centrifuged at 12,000 g (4°C) for 15 min. The supernatant was collected and mixed with isopropanol at a ratio of 1:1 and incubation for 10 min and was then centrifuged at 14,000 g (4°C) for 10 min. The supernatant was removed and 100 μl 75% ethanol was added to the tubes and mixed thoroughly. After centrifugation at 8,000 g (4°C) for 5 min, the supernatant was removed and the tubes were air-dried. cDNA was synthesized using the prime ScriptTM 1st strand cDNA synthesis kit according to the manufacturer’s instructions (Fermentas Thermo). Real-time quantification of target mRNA was performed using a SYBR premix Ex TaqTM II kit (Takara) according to the manufacturer’s instructions. Briefly, amplification was performed in a total volume of 10 μl, and each reaction contained 5 μl SYBR REmis EX TaqTM, 0.4 μl forward and reverse PCR primers, 0.2 μl ROX reference Dye II, 1 μl cDNA and 3 μl ddH2O. The real time PCR program consisted of an initial step of 10 s at 95°C, followed by 40 cycles of denaturing at 95°C for 5 s and extension at 60°C for 31 s. All measurements were performed at least three times. GAPDH was used as a housekeeping gene. The following primer sequences were used: RORγt-forward, 5-ACC TCA CCG AGG CCA TTC AG-3 and reverse, 5-TAGG CCC GGC ACA TCC TAA C-3; Foxp3-forward, 5-ATC TAC CAC TGG TTC ACA CGC AT-3 and reverse, 5-CTC CAC CCG CAC AAA GCA C-3; GAPDH-forward, 5-GGT GTG AAC CAT GAG AAG TAT GAC A-3 and reverse, 5-GTC CTT CCA CGA TAC CAA AGT TGT-3.

### Multiplex fluorochrome assay

The expression level of cytokines, such as interleukin (IL)-6, IL-10, IL-17A, IL-23, tumor necrosis factor (TNF), TGF-β and IFN-γ, were measured in serum samples, and Bio-Plex Human Cytokine Panel (Bio-Rad Laboratories) was used according to the manufacturer’s instructions. The human serum diluent kit (Bio-Rad Laboratories) was employed for purification of the samples. Identification and quantification of each specific reaction was performed with a Luminex 100 instrument (Luminex xMAP Technology, Austin). Acquisition conditions were set with a minimum of 100 beads per region. All of the raw data (median fluorescence intensity) from the bead combinations tested were analyzed using StarStation Software 2.3 (Applied Cytometry Systems). A five-parameter curve fit was used to create each standard curve to obtain the values of sample concentration. The cutoffs for minimum detectable concentration for the cytokines were as follows: 0.5 pg/mL (IL-6, IL-23), 0.6 pg/mL (IL-10, IFN-γ), 0.7 pg/mL (IL-17A) and 1.3 pg/mL (TNF). The specific antigen- and mitogen-induced cytokine secretions were calculated by subtracting the spontaneous background secretion.

### Statistical analysis

Statistical analysis was conducted with Student’s *t* test. All data analyses were performed by SPSS statistical software (version 13). Data are presented as the means ± SE. A two-tailed p-value of <0.05 was considered to be significant.

## Results

### Circulating Th17 Cells, Treg Cells, and the ratio of Th17/Treg cells in patients with URSA after immunotherapy

**Figure 1:**
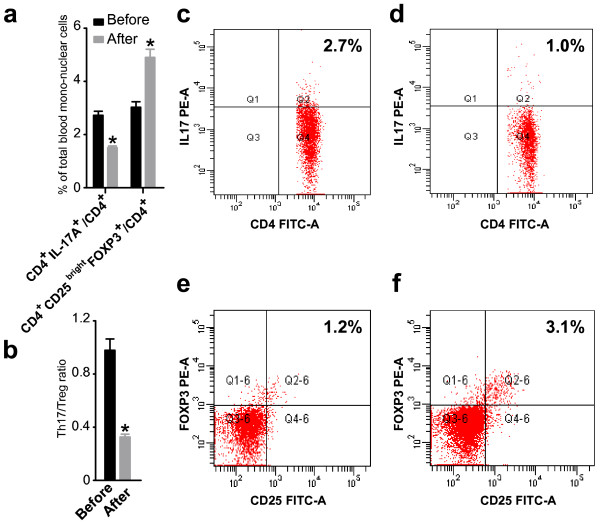
**The percentages of Th17 and Treg cells in peripheral blood mononuclear cells and the Th17/Treg ratio varied with immunotherapy.** The percentage of Th17 and Treg cells in peripheral blood mononuclear cells were detected by flow cytometry in the total patient population before and after immunotherapy, respectively. **(a)** The percentage of Th17 cells in peripheral blood mononuclear cells significantly decreased after immunotherapy (P < 0.01, paired *t*-test). Bar represents the CD4^+^/IL-17A^+^ cell frequency and values of the means ± S.E. The percentage of Treg cells in peripheral blood mononuclear cells significantly increased after immunotherapy (P < 0.01, paired *t*-test). Bar represents the frequency of CD4^+^/CD25bright/Foxp3^+^ cells and the means ± S.E. are shown. **(b)** The Th17/Treg ratio in peripheral blood mononuclear cells significantly decreased after immunotherapy (P < 0.01, paired *t*-test). Bar represent Th17/Treg ratio in total patients before and after immunotherapy and values of means ± S.E. Representative CD4+/IL-17A + flow cytometry plots in patients are shown before **(c)** and after **(d)** immunology. Representative CD4+/CD25bright/Foxp3+ flow cytometry plots in patients are shown before **(e)** and after **(f)** immunology.

**Figure 2:**
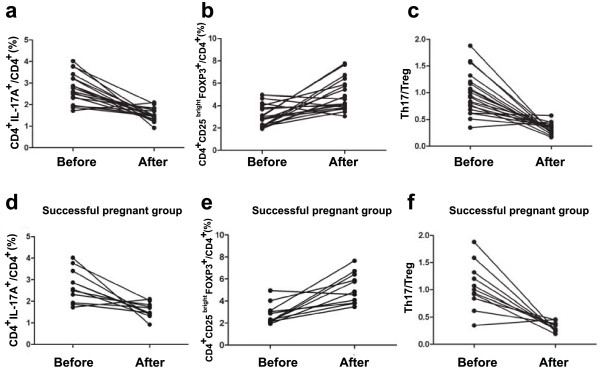
**The change of Th17 and Treg cells in peripheral blood mononuclear cells and the Th17/Treg ratio varies in total patients and patients with a successful pregnancy after immunotherapy.** The detailed variation of CD4+/IL-17A + **(a)**, CD4+/CD25bright/Foxp3+ **(b)** and Th17/Treg ratio **(c)** in each patient before and after immunotherapy is shown in line chart. The detailed variation of CD4+/IL-17A + **(d)**, CD4+/CD25bright/Foxp3 + **(e)** and Th17/Treg ratio **(f)** in each patient with a successful pregnancy after immunotherapy is shown in the line chart.

**Figure 3:**
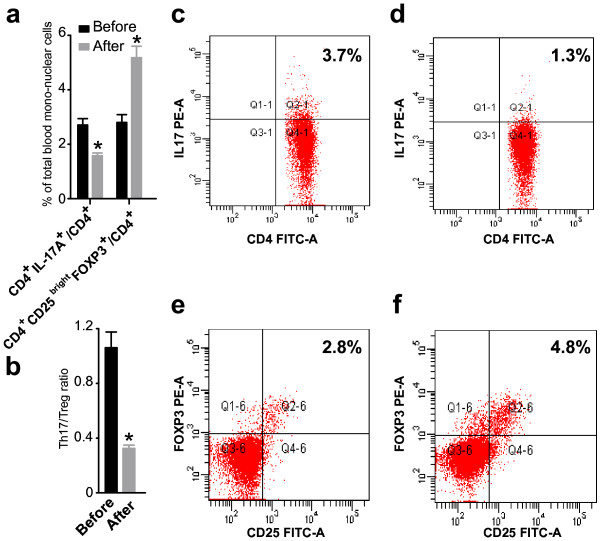
**The percentage of Th17 and Treg cells in peripheral blood mononuclear cells and the Th17/Treg ratio varies in patients with a successful pregnancy after immunotherapy.** The percentage of Th17 and Treg cells in peripheral blood mononuclear cells were detected by flow cytometry in patients with successful pregnancy after immunotherapy. To date, 13 of the 20 patients have become newly pregnant. Of the 13, the 11 patients did not miscarry (successful group). **(a)** The percentage of Th17 cells in peripheral blood mononuclear cells significantly decreased after immunotherapy. The percentage of Treg cells in peripheral blood mononuclear cells significantly increased after immunotherapy (P < 0.01, paired *t*-test). Bar represents the CD4+/IL-17A + cell frequency and the means ± S.E. are indicated **(b)** The Th17/Treg ratio in peripheral blood mononuclear cells significantly decreased after immunotherapy (P < 0.01, paired *t*-test). Bar represent the frequency of CD4+/CD25bright/Foxp3+ cells and the means ± S.E. are indicated. Representative CD4+/IL-17A + flow cytometry plots from patients with successful pregnancies were shown before **(c)** and after **(d)** immunotherapy. Representative CD4+/CD25bright Foxp3+ flow cytometry plots from patients with successful pregnancies were shown before **(e)** and after **(f)** immunotherapy.

The percentage of IL-17^+^/CD4^+^ in PBMC in the total patient population with URSA before immunotherapy was 2.73 ± 0.68%, and the percentage of this subset after immunotherapy was 1.51 ± 0.29% (P < 0.01, paired *t*-test, Figure 1). Thus, the percentage of Th17 cells significantly decreased after immunotherapy compared with that before immunotherapy (Figure 2). We quantitated the percentage of Treg cells within the CD25^bright^ cell population because only these cells represent both fide Treg in human [25,26]. As shown in Figure 3, the percentage of Tregs (CD4^+^CD25^bright^Foxp^3+^ T cells) in PBMC was significantly increased in patients with URSA following (4.94 ± 1.38%) as compared with before (3.03 ± 0.91%) immunotherapy (P < 0.01, paired *t*-test, Figure 1). Thus, the percentage of Treg significantly increased after immunotherapy (Figure 2). The mean Th17/Treg ratio in all patients with URSA before immunotherapy was 0.98 but dropped to 0.33 after immunotherapy (P < 0.05, Figure 1). Therefore, the mean Th17/Treg ratio significantly decreased after immunotherapy compared with the ratio before immunotherapy (P < 0.01, paired *t*-test), as shown in Figure 3.

### Circulating Th17 cells, Treg cells, and the ratio of Th17 and Treg cells in URSA patient with successful pregnancies following immunotherapy

To date, 13 of the 20 patients had recent pregnancies. Of these 13 patients, the pregnancy continued in 11 patients (successful group) (83.3%), while the remaining 2 cases underwent repeated abortion (unsuccessful group). Of the 11 patients with successful pregnancies, 8 patients delivered babies and the remaining 3 are still pregnant (their gestational weeks [Gw] are 20, 28 and 23). The mean percentage of CD4^+^/IL-17^+^ T cells of the successful group before immuno- therapy was 2.72% of total PBMC, and the mean percentage after immunotherapy was 1.58% (Figure 3). The percentage of Tregs (CD4^+^CD25^bright^Foxp^3+^ T cells) was significantly higher in the successful group (5.19 ± 1.38%) after than before (2.80 ± 0.95%, P < 0.01) immunotherapy (Figure 3). The mean of the Th17/Treg ratio in the successful group before immunotherapy was 1.06, and the mean ratio after immunotherapy was 0.32 (Figure 3).Thus, in the successful group, the percentage of Th17 cells (Figure 2) was significantly lower and the percentage of Tregs (Figure 2) was significantly higher after immunotherapy. The ratio of Th17/Treg cells (Figure 2) was significantly lower after immunotherapy.

### The cytokines measured in serum samples

**Figure 4:**
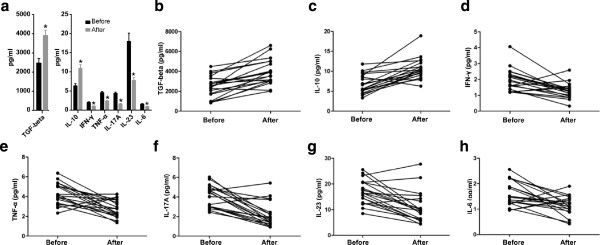
**The interleukins/cytokines were detected by multiplex fluorochrome assay in serum samples from the total patients before and after immunotherapy. (a)** The content of TGF-β, IL-10, IFN-γ, TNF-α, IL-17A, IL-23, and IL-6 in serum varied before and after immunotherapy. The detailed variation of TGF-β **(b)**, IL-10 **(c)**, IFN-γ **(d)**, TFN-a **(e)**, IL-17A **(f)**, IL-23 **(g)**, and IL-6 **(h)** in each patient before and after immunotherapy is shown in the line chart.

**Figure 5:**
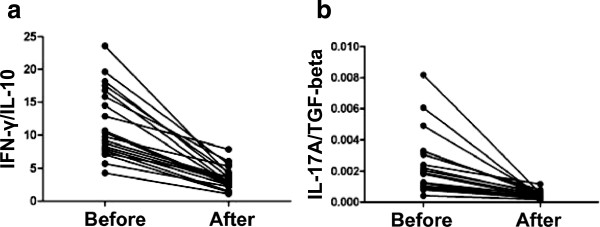
**The ratio of IFN-γ/IL-10, and IL-17A/TGF-βvaried in total patients before and after immunotherapy.** The detailed variation of the ratio of IFN-γ/IL-10 **(a)**, and IL-17A/TGF-β **(b)** in each patient is shown in the line chart.

The concentrations of Th1-type cytokines IFN-γ and TNF-α were significantly decreased in the serum of patients with URSA before immunotherapy (1.97 ± 0.69 and 4.66 ± 1.32 pg/ml) than after immunotherapy (1.03 ± 0.37 and 2.28 ± 0.96 pg/ml). The concentration of Th2-type cytokine IL-10 before immunotherapy was 6.32 ± 2.46 and after immunotherapy was 10.87 ± 4.32 pg/ml, which was a significant increase. Th17-type cytokines such as IL-6 and IL-23 were significantly decreased after immunotherapy; before immunotherapy, IL-6 and IL-23 were 1.60 ± 0.49 and 17.94 ± 9.76 pg/ml, respectively, and after immunotherapy were 0.89 ± 0.34 and 7.78 ± 3.43 pg/ml. Additionally, the concentration of TGF-β was significantly higher after immunotherapy (3909.05 ± 1248.35 pg/ml) versus before (2469.83 ± 1058.71 pg/ml). IL-17A (Fig. 4f) was significantly decreased after immunotherapy (1.57 ± 0.78 pg/ml) compared with that before immunotherapy (4.32 ± 1.40) (P < 0.01, paired *t*-test). As shown in Figure 4.Th1/Th2 balance in patients with URSA who underwent immunotherapy had been reported, and we also found that there may be a unique balance between Th1-type cytokines, such as IFN-γ, and Th2-type cytokines, such as interleukin IL-10, as shown in Figure 5. The concentration of Th17-type cytokine IL-17A was significantly lower and Treg-type cytokine TGF-β was significantly higher after immunotherapy compared with concentration before immunotherapy. The ratio of Th17/Treg cytokines was significantly lower after immunotherapy as compared with before immunotherapy (Figure 5).

### The mRNA expression of RORγt and FOXP3

**Figure 6:**
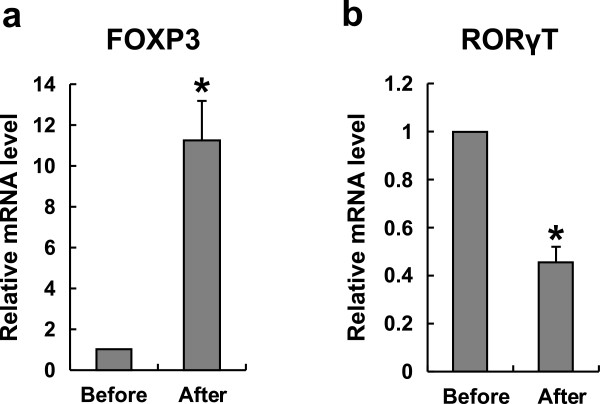
**The expression of FOXP3 and RORγT mRNA varied before and after immunotherapy in URSA patients. (a)** The mRNA level of FOXP3 was increased significantly in the total patients after immunotherapy (P < 0.01, students *t*-test). Bar represent the values of 2-ΔΔCt. **(b)** The mRNA level of RORγT was decreased significantly in the total patients after immunotherapy (P < 0.01, students *t*-test). Bar represents the values of 2-ΔΔCt.

The most important transcription factor for the differentiation of naïve T cells into Tregs is the forkhead box P3 (Foxp3) [24]. Similarly, retinoic acid–related orphan receptor t (RORγt) [23] is a crucial transcription factor for the development of Th17 cells. The mRNA expression of Foxp3 was significantly increased after immunotherapy. Meanwhile, the mRNA expression of ROR γ t was significantly decreased after immunotherapy, as shown in Figure 6.

## Discussion

Several studies characterizing immunotherapy in URSA patients reported that the outcome of subsequent pregnancies was apparently improved by injection of paternal or a third party’s leukocytes [14-18], although the findings of Ober C et al. [27] suggested this treatment is ineffective. The efficacy of immunotherapy might be related to the immune response by allogeneic lymphocytes and might not be simply a placebo effect. From a worldwide meta-analysis study, it has been indicated that immunization may be highly effective, although only for a small number of patients with URSA [28,29]. In our center, 80-90% of URSA patients who underwent immunotherapy successfully delivered a baby. Although immunotherapy was considered possibly efficacious, the underlying mechanisms have not yet been fully elucidated.

The Th1⁄Th2 balance in circulating T cells has been reported to be shift in favor of Th2 dominance during normal pregnancy, whereas the Th1 dominance has been found to be associated with reproductive failures such as URSA [30,31]. Although Wilczynski JR et al. reported that Th1/Th2 bias was not correlated with subsequent pregnancy success in the patients who accepted paternal lymphocyte immunization (PLI) [32], most related studies suggested that Th1⁄Th2 imbalance plays an important role in URSA and is a target for treatment of PLI [19-21]. Tomokazu Yokoo et al. [20] and Qiu L et al. [21] reported that immunotherapy with the husband’s mononuclear cells could induce a dominant state of Th2 cells in URSA patients. Th1 type cytokines, such as TNF-α and IFN-γ, are detrimental to pregnancy at high concentrations [21]. In the present study, we also found that TNF-α and IFN-γ were significantly decreased after immunotherapy. Additionally, Th2-type cytokine IL-10 was significantly increased after immunotherapy [21]. The induction of Th2 bias might be correlated with the successful continuation of pregnancy in patients with URSA who undergo immunotherapy with lymphocytes derived from the baby’s father.

Several studies support our findings that the proportion of CD4^+^CD25^+^ Treg cells in peripheral blood lymphocytes was significantly lower in URSA women than in normal (non-pregnant) control subjects, which suggests that CD4^+^CD25^+^ Treg cells might contribute to the mechanisms mediating maternal immune tolerance of conceptus antigens, and therefore, Treg cells might contribute to the maintenance of pregnancy [25,26]. The reciprocal developmental pathway for the generation and the opposing effects of Th17 and Treg cells may affect the balance between these cell subsets in patients with URSA [4,5,12]. Investigators suggested a potentially deleterious role for Th17 cells in pregnancy [12,13]. Our previous data [13] also implicated that the imbalance between Th17 and Treg cells may play an important role in the pathogenesis of URSA. Hui Yang et al. [22] demonstrated that lymphocyte immunization significantly increased the percentage of Tregs in peripheral blood in women with URSA, contributing to improved pregnancy outcome. The data suggested that Tregs may be a novel target in URSA therapy.

Progesterone is required for the establishment and maintenance of pregnancy, and the link between progesterone and the immune system is also established. Several reports suggested that the immunological effects of progesterone are mediated by progesterone-induced blocking factor (PIBF). Through the Il-4 receptor, PIBF induces a Th2-dominant cytokine reponse with increased production of IL-3, IL-4 and IL-10 [33,34]. The treatment of progesterone in Th1-like T cells led to increased expression of IL-4 mRNA and production [35]. These results indicated that PIBF might play a role in regulation of Th1/Th2 balance during lymphocyte immunotherapy of URSA.

In present study, we first analyzed Th17 cells, Treg cells, and the Th17/Treg balance in URSA patients following lymphocyte immunization. Subsequently, we detected a significant decrease in Th17 cells, a significant increase in Treg cells in PBMCs, and a significant decrease in the Th17/Treg ratio in URSA patients after immunotherapy. Furthermore, this phenomenon was reinforced by the demonstration that URSA patients could have a successful subsequent pregnancy after immunization therapy. It has been reported that increased IL-23 or IL-6 production and decreased TGF-β production might cause an increase in Th17 cells and a decrease in Treg cells in the uterus [10,12,36,37]. We detected the Th17-related soluble cytokines, such as IL-6, IL-17A, and IL-23 in serum, and found that levels of these cytokines were significantly decreased after immunotherapy. Similarly, levels of Treg-associated cytokines IL-10 and TGF-β were significantly higher after immunotherapy. Tregs exert their function partly through secretion of the anti-inflammatory cytokines IL-10 and TGF-β. Additionally, TGF-β can promote Foxp3 expression by inducing Treg differentiation from CD4+/CD25 + T cells [38-42]. This is in accordance our data that the expression of Foxp3 mRNA was higher after immunotherapy compared with before immunotherapy. Conversely, the expression of ROR γ t mRNA was lower after immunotherapy.

## Conclusions

In this context, we initially analyzed the Treg cells bias against Th17 cells and whether it may play a role in the maintenance of pregnancy. It is possible that immunotherapy affects the Th17 and Treg cell balance in patients. Therefore, up-regulation of the Treg population and down-regulation of the Th17 population might contribute to the outcome of pregnancy. The data suggest that regulating the balance of Th17/Treg may be a novel target in URSA therapy. The limitation of this study is the number of samples is small, and further study is required.

## Abbreviations

FITC: Fluorescein isothiocyanate; Foxp3: Forkhead box p3; Gw: Gestational weeks; IFN-γ: Interferonγ; IL: Interleukin; PBMC: Peripheral blood mononuclear cells; PBS: Phosphate buffered saline; PMA: Phogrbol12 -myristate13-acetate; q PCR: Quantitative real-time PCR; ROR γ t: RETINOID-related orphan receptor gamma; RSA: Recurrent spontaneous abortion; TGF-β: Transforming growth factor-β; Th1: T helper 1 cells; Th2: T helper 2 cells; Th17: T helper 17 cells; TNF-α: Tumor necrosis factor; Treg: Regulatory T cells; URSA: Unexplained recurrent spontaneous abortion.

## Competing interests

The authors declare that they have no competing interests.

## Authors’ contributions

LW and LHL conducted the experiment and data collection, participated in the design of the study, and drafted the manuscript. YXZ and QL participated in the processing of the experiment and collection data. BX performed the statistical analysis and the diagramming. GXZ and HBL conducted the interpretation of the data and there vision for important intellectual content and grammar. YSL conceived of the study, participated in its design and coordination, and helped to draft the manuscript. All the authors read and approved the final manuscript.

## Supplementary Material

Additional file 1: Table S1 Clinical characteristics of the patients. Click here for file
