# Supplementary material for: Alteration of Th17 and Treg cells in patients with unexplained recurrent spontaneous abortion before and after lymphocyte immunization therapy
Source: Reprod Biol Endocrinol. 2014 Aug 3;12:74. doi: 10.1186/1477-7827-12-74 (PMC4237930; doi:10.1186/1477-7827-12-74)
Supplement: Additional file 1: Table S1 — Clinical characteristics of the patients. [file 1477-7827-12-74-S1.doc]

| **Supplementary Table S1. Clinical characteristics of the patients.** | |
| --- | --- |
| **Deviation age** | 28.6 ± 0.15 |
| **Number of miscarriages** | 4 (range, 3–7) |
| **Donor (husband)** | 12 |
| **Donor (third party)** | 8 |
| **Mullerian anomaly** | Negative |
| **Hormonal deficiency** | Negative |
| **Metabolic disorder** | Negative |
| **Infectious disease** | Negative |
| **Autoimmune abnormalities** | Negative |
